# Pancreatic carcinoma cells colonizing the liver modulate the expression of their extracellular matrix genes

**DOI:** 10.18632/genesandcancer.179

**Published:** 2018-05

**Authors:** Khamael M.K. Al-Taee, Michael Zepp, Irina Berger, Martin R. Berger, Hassan Adwan

**Affiliations:** ^1^ Toxicology and Chemotherapy Unit, German Cancer Research Center, Heidelberg, Germany; ^2^ Institute of Pathology, Klinikum Kassel, Mönchebergstraße, Kassel; ^3^ German University of Cairo, Cairo, Egypt

**Keywords:** pancreatic ductal adenocarcinoma, ASML cell line, liver metastasis, mRNA and miRNA expression, extracellular matrix

## Abstract

Liver is the main target of pancreatic ductal adenocarcinoma (PDAC) metastasis. Here, a rat model was used for analysing gene expression modulations during liver colonization. ASML PDAC cells were injected to isogenic rats and re-isolated at various stages of liver colonization for RNA isolation or re-cultivation. Microarrays were used for analysing mRNA and miRNA profiles of expres­sion. The results were partially confirmed by (q) RT-PCR and western blot. Selected genes were knocked down by siRNA transfection and the resulting cell behaviour was analysed. The ratio of up- and down regulated genes decreased from 20:1 (early stage) to 1.2:1 (terminal stage). Activation of cancer relevant gene categories varied between stages of liver colonization, with a nadir in the intermediate stage. The cells' environment triggered up to hundredfold changed expression for collagens, matrix metalloproteinases and chemokines. These modulations in mRNA expression were related to respective changes at miRNA levels. Gene expression knockdown of Mmp2 and Ccl20, which were highly modulated in vivo, was correlated with reduced prolif­eration and migration in vitro. Thus, target genes and temporal alterations in expression were identified, which can serve as basis for future therapeutic or diagnostic purposes.

## INTRODUCTION

Pancreatic ductal adenocarcinoma (PDAC) is one of the most lethal forms of cancer in Western countries, as it is mostly diagnosed at advanced stage with distant metastases [1–3]. For anatomic and organ specific reasons, the liver is the main target organ of PDAC metastasis and can be affected­ by hematogenic spread, but also by local invasion of cancer cells, growth along the nerve sheaths [4], as well as lymphatic spread. For these processes, genes need to be activated, which confer the respective properties and thus enable the cancer cells to metastasize [5, 6].

Identification of these genes will contribute to better understanding the mechanisms and signalling cascades, which are involved and will be basis for detecting new approaches in the prevention and treatment of PDAC. Hematogenic metastasis is based on sub-processes, like dissociation from the primary, intravasation, survival in the blood stream, extravasation, adaptation to a new environment, and colonization of a distant organ [7]. It is well established, however, that metastasis­ is a very inefficient process, presumably because only a minority of cells dissociating from the primary have gained all the functions essential to successfully complete metastasis [8, 9]. In order to concentrate on disseminated cells with full metastatic potential, we have chosen to work with a rat model, which mimics the final stage of PDAC liver metastasis, i.e. liver colonization [10]. Our assumption was that those cancer cells, which have started to colonize an organ, have gained most of the afore-mentioned functions and properties necessary for liver colonization. Therefore, we have re-isolated PDAC cells, which had succeeded to grow in rat liver. Specifically, we hy­pothesized that those PDAC cells, which started to grow, will have modulated the expression of genes, which are beneficial for their survival. In a first survey, we have described the overall changes in gene expression and differentiated between phases of initial and advanced growth of ASML rat PDAC cells in the liver of isogenic rats. Furthermore, we identified a number of gene products, which were modulated only in the initial phase of liver colonization [11].

Subsequently, we hypothesized that liver and invading tumour cells will influence each other during­ liver colonization. In this regard the liver extracellular matrix (ECM) would be the first contact­ for invading tumour cells, and genes as well as epigenetic factors allowing the tumour cells to influence the ECM could be decisive for their successful growth within the new environment. The ECM is a well-defined structure that is composed of secreted molecules, which comprise, among others, structural proteins, non-collagen glycoproteins, matrix metalloproteinases, glycopeptidases,­ growth factors, and chemokines. Due to its composition, the ECM is involved in many pathophysiological processes such as wound healing, fibrosis, tumour invasion, metastasis and angiogenesis [12–15].

In the current article we have expanded our former work and report on all stages of liver colonization­ as well as all genes modulated during these processes, with special emphasis given to genes contributing to the ECM. In addition, we have gathered information on micro RNA (miRNA) modulation during all colonization stages and have linked these miRNAs with those genes, which they regulate. Finally, based on our experimental setting, involving an initial period of *in vivo* growth and subsequently in cell culture, we expected to differentiate between genes which are vital for *in vivo versus in vitro* conditions.

## RESULTS

### Modulation of metastasis related genes from re-isolated ASML cells

**Figure 1:**
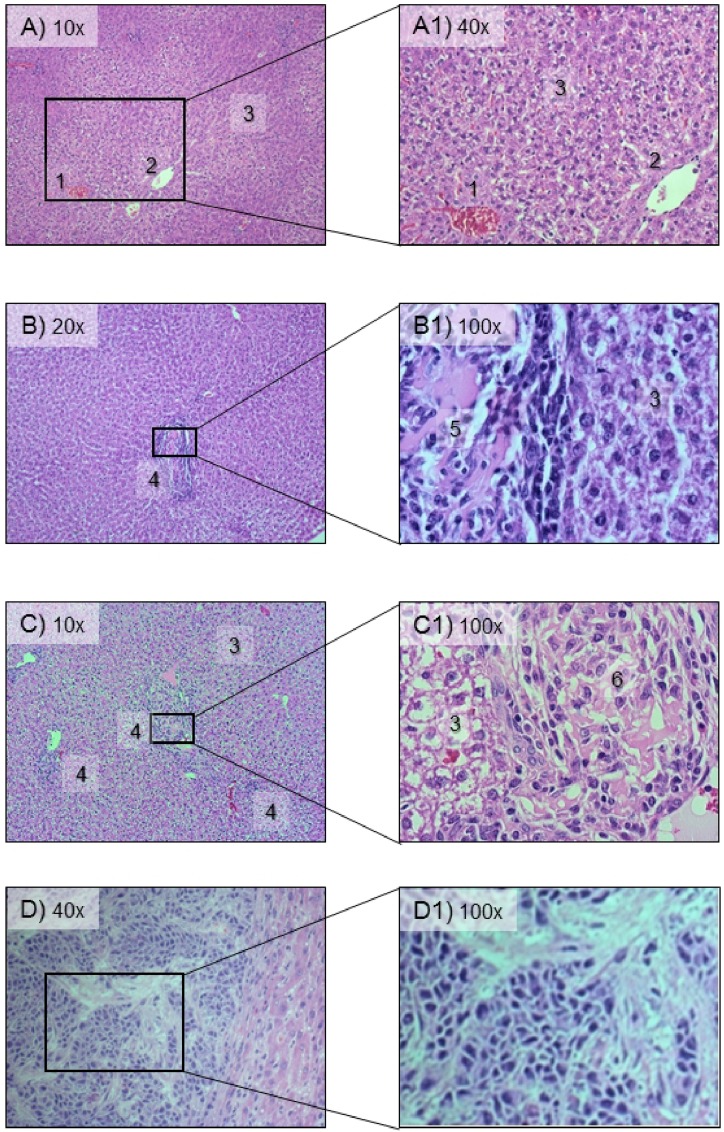
Histopathology of rat livers after intraportal injection of ASML PDAC cells The sections­ were stained by hematoxylin & eosin and examined for the presence and number of invad­ing ASML cells. **A.** The upper row shows a normally appearing liver excised at one day after tumor cell inoculation, the structures indicated by numbers correspond to a central vein (1), a portal tract (2), and normal hepatocytes (3). **B.** The middle row shows a liver, which was excised at three days after tumor cell inoculation, which is invaded by ASML lesions containing less than 20 tumor cells, as indicated by (4) and (5). These two periods after tumor cell inoculation were termed ‘initial stage'. **C.** The third row corresponding to liver being excised­ at 6 days after tumor cell inoculation shows an increased number of ASML lesions, with more than 20 tumor cells per lesion (6). This period was termed ‘intermediate stage'. **D.** The bottom row corresponds to liver, being excised at 15 days after tumor cell inoculation, which shows gross infiltration of liver tissue by ASML cells as well as macroscopic visible tumor nodules. This period was termed advanced stage.

Modulations in the expression profile of 30,508 rat genes were analysed by mRNA microarray to establish the correlation between liver colonization and ASML cell gene expression. The gene expression profiles of cells isolated at days 1 and 3 after tumour cell inoculation were classified as early stage, because no tumour was visible. ASML cells isolated at day 6 were considered as intermediate­ stage, here ASML cells showed signs of infiltrative growth into rat liver, which was visible as white spots of 1-2 mm in diameter. The time of 15 days after tumour cell inoculation was defined as advanced stage because the ASML cells colonized about 50% of the rat liver and the tumour spot size had increased to ~7 mm in diameter. The terminal stage was considered when ASML cells had infiltrated almost the whole rat liver (21 days post injection). Histopathologic evaluation by H&E stain was performed for early and intermediate periods after tumour cell inoculation for differentiating between the three stages, which are not or just barely visible by naked eye. The results are shown in Figure 1. At day one after tumour cell inoculation, no tumour cells were visible by H&E stain (Figure 1A, top row). After three days, there was tumour cell infiltration, but the size of individual lesions corresponded to less than 20 tumour cells; in ad­ dition, these lesions were surrounded by inflammatory cells (Figure 1B, second row). Thus the early stage is characterized by few tumour cells, which potentially evoke a host reaction. In the intermediate stage, after six days, the number of tumour lesions had increased; they showed more than 20 tumour cells per le­sion but were still below a size of 2 mm in diameter, thus corresponding to micro-metastases (Figure 1C, third row). Advanced and terminal stages on days 15 and 21 corresponded to macro-metastases with lesions larger than 2 mm in diameter (for advanced stage see Figure 1D, bottom row).

**Figure 2:**
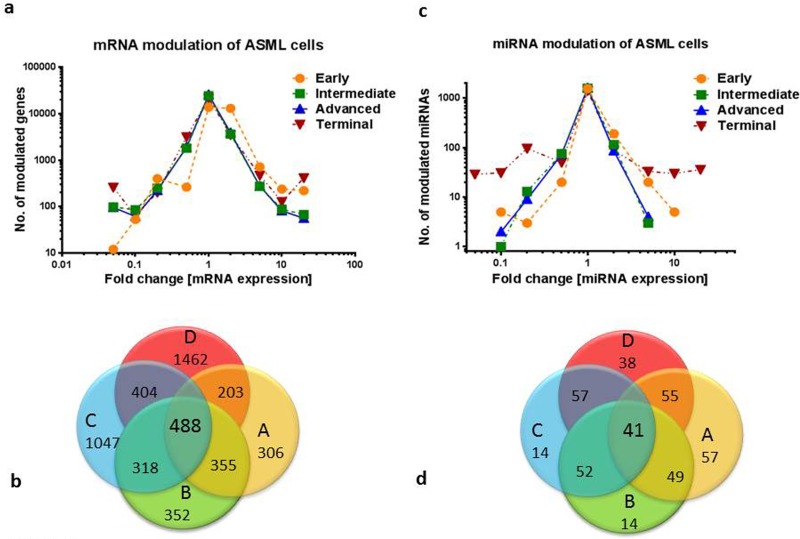
Overview of mRNA and miRNA modulation in ASML cells during rat liver colonization­ The number of mRNAs a. or miRNAsc.withmodulatedtranscriptionisshownversusrespectivefoldchangesinexpression,withtheinvitroexpressionofASMLcellssettounity. Venn diagrams showing the number of at least twofold modulated cancer-associated mRNAs b. or miRNAs d. for each stage, as well as the number of overlapping mRNAs / miRNAs during liver colonization. Stages of colonization are classified as early (A), intermediate (B), advanced (C), and terminal (D) stages.

When analysing the microarray results, the mRNA profile of cells isolated during the early stage indicated that 14,215 genes (46.5%) showed ≥ 2fold increased expression, but only 728 genes (2.3%) exhibited ≥ 2fold decreased expression. The expression profile of the intermediate stage showed 4,057 genes (13.2%) that were ≥ 2 fold up-regulated and 2,252 genes (7.3%), which were ≤ 0.5fold down-regulated. In the advanced colonization stage 4,310 genes (14.1%) showed ≥ 2fold up-regulation and 2275 genes (7.4%) were ≤ 0.5fold down-regulated. In the final stage 4,530 genes (14.8%) were ≥ 2fold up-regulated and 3,697 genes (12.1%) were ≤ 0.5fold down-regulated. The ratio between up and down regulated genes decreased from 19.5:1 (early stage) to 1.2:1 (terminal stage). A breakdown of these changes is shown in Figure 2a.

As Figure 2a also reveals, the number of genes with more than 20fold change in expression was most marked in the final stage. When considering cancer associated genes only, as defined by IPA analysis, a rising number of genes were involved. Of special interest were those genes, which showed altered expression in two subsequent or in all stages, because they might be involved­ in the changes described as cancer progression. Figure 2b gives a summary of all cancer related genes with expression changes as well as the number of genes overlapping between two or all stages.

### Gene functions and rat liver colonization stages

The concerted reaction of genes, which contribute to the same function, was measured by both, a p-value and an activation z-score. Interestingly, unexpected profiles of gene expression were found during liver colonization for a variety of function categories.

‘Migration - cellular movement' was increased in early and terminal stages, but not increased or even decreased in intermediate or advanced stages. ‘Cell proliferation' was likewise increased in early and terminal stages but not increased or even decreased in intermediate or advanced stages. ‘M - Phase of tumour cell line’ was increased during early, intermediate and final stages but not during the advanced stage. ‘Invasion of tumour cells’ was decreased during early and advanced stages but not changed during intermediate and terminal stages. In contrast to the previous processes,­ ‘vascularization and angiogenesis’ was increased only in the terminal stage. For comparison,­ ‘synthesis of reactive species (ROS)’ was increased during all stages except the intermediate stage (Table 1).

**Table 1:** Some categories and related diseases or functions annotations with significantly altered expression

Categories	Cellular movement, haematological system development and function, immune cell trafficking, inflammatory response				Cellular development, cellular growth cell cycle &proliferation			Cancer, organis-mal injury & abnormalities, tumour morphology	Cardiovascular system development & function, organismal development		Free radical synthesis
Diseases or functions annotation	Movement of mononuclear leukocytes	Migration of phagocytes	Migration of tumour cells	Homing of tumour cells	Cell proliferation of tumour cell	M phase of tumour cell	Proliferation of fibroblast	Invasion of tumour	Vascularisation	Angio-genesis	Reactive oxygen species
**No of genes ^a)^**	121	80	46	185	44	26	93	51	26	280	142
**Early stage ^b)^**	increased ^c)^	increased	increased	increased	increased	increased	increased	decreased **^c)^**	not significant	not significant	increased
**p-Value**	4,96E-23	6,82E-20	1,54E-04	1,35E-14	5,51E-25	5,68E-10	7,67E-19	2,04E-14	3,51E-14	-	8,94E-09
**Activation z-score ^d)^**	2,812	2,563	2,634	4,349	4,574	2,123	2,436	−2,156	1,737	−	2,544
**Intermediate stage ^b)^**	not significant	not significant	decreased	decreased	decreased	increased	not significant	not significant	decreased	not significant	not significant
**p-Value**	−	−	3,64E-17	2,66E-20	2,63E-13-	1,07E-12	−	−	2,63E-13	4,51E-32	−
**Activation z-score ^d)^**	−	−	−2,221	−2,815	2,137	2.486	−	−	−2,137	−1,307	−
**Advanced stage ^b)^**	increased	not significant	decreased	decreased	decreased	not significant	not significant	decreased	not significant	not significant	increased
**p-Value**	2,88E-10	−	8,44E-13	7,97E-17	5,19E-12-	−	−	1,52E-10	8,51E-11	6,53E-27	3,71E-09
**Activation z-score ^d)^**	2,095	−	−2,581	−2,323	2,319	−	−	−2.301	−1,445	6,53E-27	2,337
**Terminal stage ^b)^**	increased	increased	increased	increased	increased	increased	increased	not significant	increased	increased	increased
**p-Value**	5,40E-30	3,21E-23	1,66E-14	1,84E-06	1,88E-07	8,14E-14	1,69E-14	−	1,25E-14	8,74E-25	1,06E-04
**Activation z-score ^d)^**	3,614	3,623	2,979	2,770	2,095	−2,783	2,319		2,194	2,224	2,648

^a)^ Number of genes listed under this category in Ingenuity pathway analysis

^b)^ Stages (early, intermediate, advanced and terminal) of liver colonization by rat ASML PDAC cells

^c)^ Increased / decreased describes a significant change in mRNA expression of the respective category of genes.

^d)^ An activation z score ≥ 2 indicates that a function is significantly increased whereas a z-score ≤ 2 specifies a significantly decreased function.

### Micro RNA modulation during liver colonization

Concomitantly, miRNA expression was studied during all stages of liver colonization. Altogether,­ 1,776 miRNAs were tested by chip array, but in the early stage there were just 190 miRNAs,­ which were up-regulated ≥ 2fold, and 5 miRNAs ≥ 10fold. Likewise, there were 20 miRNAs,­ which were down-regulated ≥ 2fold, 3 miRNAs, which were down-regulated ≥ 5fold and only 5 miRNAs ≥ 10 fold. In the intermediate, advanced and terminal stages, the number of modulated­ miRNAs generally increased, with a maximum at the terminal stage as shown in Figure 2c. Figure 2d shows the number of overlapping miRNAs that may play a role in the transition from one stage to another.

### Effect of liver environment on metastasis related gene expression

**Figure 3:**
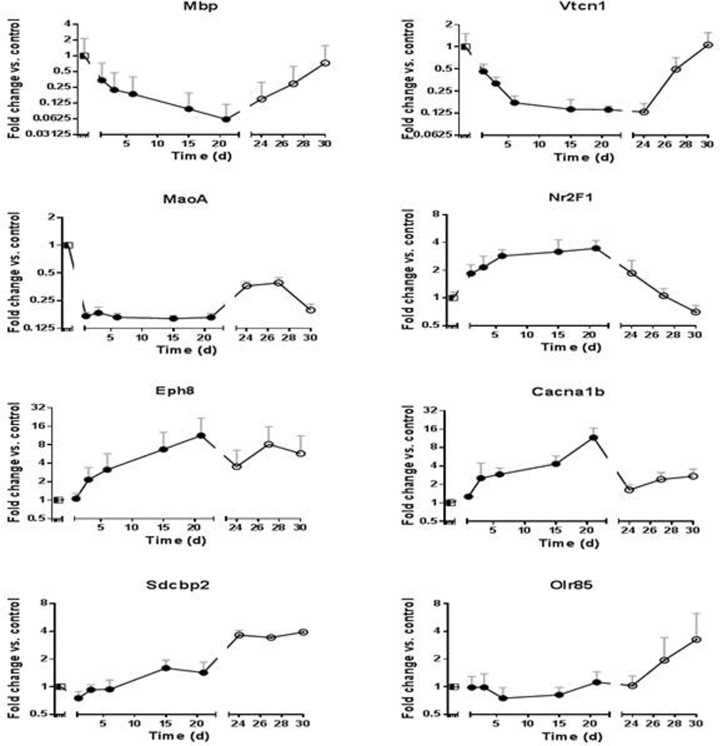
mRNA expressions pattern during all stages of liver colonization (days 1, 3, 6, 15, 21) as well as during *in vitro* growth of re-isolated ASML PDAC cells (days 24, 27, 30) The expression­ level of ASML cells growing *in vitro* was set to unity. **a.** Myelin basic protein (Mbp) **b.** V-set domain containing t-cell activation inhibitor 1 (Vtcn1) **c.** monoamine oxidase A (Mao-A), **d.** Nuclear receptor subfamily 2 group f member 1 (Nr2f4), **e.** Eph Receptor A8 (Epha8), **f.** Calcium voltage-gated channel subunit alpha1 B (Cacna1b), **g.** Syndecan binding protein 2 (Sdcbp2), (h) Olfactory receptor 85 (Olr85).

During liver colonization, many ASML cell genes showed a specific change in expression as compared to that found *in vitro*. These changes were categorized into diverse types of mRNA responses. The genes shown below were selected for their clear differences in gene expression modulation, which renders them to prototypes of distinct expression profiles. For example, the genes ‘Myelin basic protein’ and ‘V-set domain containing t-cell activation inhibitor 1′ (Mbp and Vtcn1; Figure 3a, 3b) showed a continuously decreased expression­ during the whole period of liver colonization. Nevertheless, after their transfer to *in vitro* conditions there was a return to the former level of expression as observed in cell culture. Based on these antidromic patterns / opposing trends observed during and after liver colonization, it is conceivable that these genes reacted according to conditions set by their environment. In addition, there were genes, which showed other types of expression modulation, i.e. low levels during liver colonization­ but high levels *in vitro*, as for monoamine oxidase A (Mao-A; Figure 3c) or increasing levels during liver colonization and decreasing levels *in vitro* as shown for Nuclear receptor subfamily 2 group f member 1 (Nr2F1; Figure 3d). By contrast, Eph Receptor A8 (Epha8, Figure 3e) and Calcium voltage-gated channel subunit alpha1 B (Cacna1b; Figure 3f) showed increasing­ expression during the time of liver colonization and remained at increased level after their re-isolation *in vitro*. Finally, Syndecan binding protein 2 and Olfactory receptor 85 (Sdcbp2, Olr85; Figure 3g, 3h) did not show signifi­cant changes in expression but increased distinctly *in vitro*.

### Effect of liver environment on miRNA expression in ASML cells

**Figure 4:**
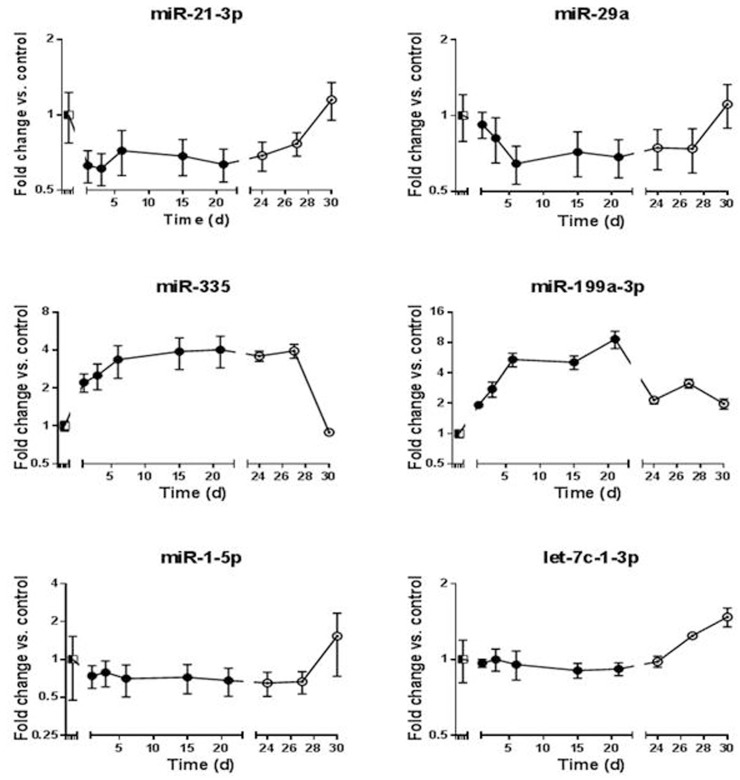
miRNA expression pattern during all stages of liver colonization (days 1, 3, 6, 15, 21) as well as during *in vitro* growth of re-isolated ASML PDAC cells (days 24, 27, 30) The expression­ level of ASML cells growing *in vitro* was set to unity. **a.** miR-21-3p, **b.** miR-29a, **c.** miR-335, **d.** miR-199a-3p, **e.** miR-1-5p, **f.** miR-let7c-1-3p.

As for mRNAs, effects of the surrounding environment became obvious by comparing expression levels during colonization and those observed thereafter *in vitro*. miR-21-3p expression, which is down-regulated in several human cancers, was reduced in rat liver but re-amplified to a high ex­pression level *in vitro* (Figure 4a). A similar picture was found for miR29a, (Figure 4b). A con­ trasting profile was found for miR-335 and miR-199-3p, which showed significantly increased expression during liver colonization but decreased expression *in vitro* (Figure 4c, 4d). Finally, some miRNAs showed no significant alteration during liver colonization but an increased expression *in vitro*, as for miR-1-5p, and miR-let-7c-1-3p (Figure 4e, 4f).

### Interplay between mRNAs and miRNAs during liver colonization

Messenger RNA transcription leading to protein expression is regulated by the presence of miRNAs­. We tried to correlate miRNA levels detected during liver colonization with mRNA expression­ levels of dependent genes (Table 2).

**Table 2:** Interplay between miRNAs and corresponding mRNAs

Symbol	Early stage	Intermediate stage	Advanced stage	Terminal stage	Symbol	Early stage	Intermediate stage	Advanced stage	Terminal stage
miR-146a-5p (GAGAACU)^a^	4.20	−1.22	−1.30	2.53	CXCR4^b^	2.30	2.73	3.26	11.92
					COL13A1^c^	2.34	2.447	1.27	−1.09
miR-23a-3p (UCACAUU)	2.70	1.82	1.44	2.38	CXCL12^b^	2.70	1.78	1.15	11.74
miR-29b-3p (AGCACCA)	−2.50	−2.86	−4.76	−3.03	COL1A1^b^	2.90	1.02	1.06	58.59
					COL1A2^b^	5.30	1.14	2.01	340.33
					COL3A1^b^	8.50	16.16	3.47	37.16
					COL4A1^b^	4.20	2.45	1.17	131.04
					COL4A2^b^	2.30	1.55	1.00	6.58
					COL5A3^b^	1.90	1.07	1.17	2.03
					COL5A2^b^	0.29	0.14	0.12	0.38
let-7a-5p (GAGGUAG)	2.91	2.48	2.78	−5.00	COL1A2^b^	5.30	1.14	2.01	340.33

^a^ Seed region of miRNA

^b^: Site of function: plasma membrane

^c^: Site of function: extracellular space

Interestingly, there was no mono-directional association, as shown by miR146a-5p, the expres­sion of which was related to moderate increases in Cxcr4 and Col13a1 mRNA levels during early to advanced stages, but to divergent effects in the terminal stage, as indicated by a 12fold increase­ of Cxcr4 and a minimal decrease of Cola13 a1. In the case of miR23a-3p, modestly in­creased levels were correlated with almost identical increases in Cxcl12 mRNA during early to advanced stages, but an un-proportional increase in Cxcl12 mRNA during the terminal stage.

The reduced expression of miR29b-3p was related to increased mRNAs levels of various collagens­ (Col1a1, Col1a2, Col3a1, Col4a1, Col4a2, Col5a2, and Col5a3). Again, there was an un-proportional increase in the level of these mRNAs in the terminal stage of liver colonization. A partial explanation is given by the expression of miRNA let-7a-5p. Here, modestly increased levels­ during early to advanced stages were followed by a distinct drop in the terminal stage. As both, miR-29b-3p and let-7a-5p, regulate Col1a2, the final decrease of both miRNA levels may at least in part explain the terminally distinct increased expression of Col1a2 mRNA.

### Extracellular matrix genes, inflection of expression during liver metastasis

The analysis of miRNA and mRNA expression from ASML cells had suggested that the environment­ influences the expression profile of selected genes. Extracellular matrix genes include several gene families, which constitute or alter the ECM [14, 15].

An overview on ECM genes, which were modulated, is given in Table 3. From the group of collagens, the major structural proteins of ECM, 16 of 39 genes (41%) were significantly up-regulated in mRNA expression, and 13 (33%) were down regulated (for details see suppl. Figure 1a, 1b). From the group of laminins, only three of eight genes showed significantly altered mRNA expression­. These included Lamc2, Lama5 and Lamb2 (suppl. Figure 1c). Interestingly, elastin showed more than 2fold increased expression during early to advanced liver colonization but was expressed at normal level during the final stage. A different profile was found for brevican, which was increased significantly only during the early stage of colonization (supplementary Figure 1d). Expression of some glycoproteins is shown in suppl. Figure 2a. Vitronectin (Vtn) is a constituent of ECM, which can bind to integrin αvβ_3_ and promote cell adhesion. In this microarray, Vtn was highly up-regulated initially and during the final stage. From the fibrillins, fibrillin1 was down-regulated, while fibrillin 2 was not significantly changed in expression. With regard to its inter­action with integrins, the glycoprotein fibronectin (Fn) is a related constituent of ECM. Fn was regulated in a way mimicking that of Vtn, i.e., it was highly up-regulated during initial and final stages of liver colonization. In contrast, the fibronectin receptor integrin α5 was slightly down-regulated during all stages. Tenascin, moreover, did not show any significant modulation­. In contrast, Nidogen1/entactin showed significantly reduced expression during all stages of liver colonization. The hyaluronan and proteoglycan link proteins, Hapln1 and Hapln4, didn't show significant changes in expression, but Hapln2 and Hapln3 were significantly decreased in expression. Hyaluronan binding protein 4 (Habp4) was significantly reduced only in the advanced stage (supplementary Figure 2b).

**Table 3:** Overview of extracellular matrix components of ASML-PDAC cells showing alterations in mRNA expression during the course of liver colonization

Extracellular matrix component	Showing upregulation^a)^	Showing downregulation^a)^	For details see
Collagens (Col)	Col1a1^*b)^, **Col2a1**^b)^, Col3a1^*^, Col4a1^*b)^, **Col4a2**^b)^, **Col4a3**, **Col4a4**, Col5a1^b)^, Col5a3^b)^, Col6a1^*b)^, Col6a2^*b)^, **Col9a1**^b)^, **Col9a2**, **Col10a1**^b)^, Col11a1^*b)^, **Col11a2**, **Col13a1**^b)^, **Col15a1**^b)^	**Col1a1**^b)^, Col2a1^b)^, Col4a1^b)^, Col4a2^b)^, **Col4a5**, **Col5a1**^b)^, Col5a2^*^, **Col5a3**^b)^, **Col6a1**^b)^, Col6a2^b)^, **Col7a1**, Col8a1^*^, Col9a1^b)^, Col10a1^b)^, Col11a1^b)^, Col12a1^*^, Col13a1^b)^, **Col14a1**, Col15a1^b)^, **Col16a1**, **Col17a1**, **Col22a1**, **Col24a1**, **Col27a1**	Suppl. Figures 1a and 1b
Laminins (Lam)	Lama5^b)^, Lamb3^b)^, Lamc1^b)^, **Lamc2**, Lamc3^b)^	Lama1, Lama2, **Lama5**^b)^, **Lamb2**, Lamb3^b)^, Lamc1^b)^, Lamc3^b)^	Suppl. Figure 1c
Miscellaneous proteins	**Elastin**, **Bcan**^b)^	**Bcan**^b)^	Suppl. Figure 1d
Glycoproteins	Vitronectin^*^, Fibronectin1^*^, Fibrillin1^b)^, **Fibrillin2**^b)^,	**Ribronectin receptor**, **Fibrillin1**^b)^, Fibrillin2^b)^, Nidogen 1^*^/Entactin^*^ Tenascin R	Suppl. Figure 2a
Hyaluronan and link proteins (Hapln)	Hapln1^b)^, Hapln4	Hapln1^b)^, **Hapln2**, **Hapln3**, Hapln4^b)^, **Habp4**	Suppl. Figure 2b
Proteoglycans	Perlecan^b)^	**Aggrecan**, Neurocan, Versican^*^, Perlecan^b)^	Suppl. Figure 2c
Matrix metalloproteinases (MMP) / Tissue inhibitors of metalloproteinases (Timp)	Mmp-2^*^, **Mmp-7**, Mmp-12^*^, **Mmp-14, Mmp-17**, **Mmp-23**	**Mmp-11**, **Mmp-19**, **Mmp-24**, **Timp-1**	Suppl. Figure 2d
A disintegrin and metalloproteinase (ADAM)	**Adam18**, **Adam20**	**Adam1a**, **Adam6**, **Adam7**, **Adam9**, **Adam10**, **Adam15**, **Adam17**, **Adam19**, **Adam22**, **Adam23**, **Adam28**, **Adam30**, **Adam33**, **Adam34**	Suppl. Figure 2e
A disintegrin and metalloproteinase with thrombospondin motifs (ADMTS)	**Adamts1**, **Adamts2,** Adamts9^b)^	**Adamts4**, **Adamts5**, **Adamts9**^b)^, **Adamts12**, **Adamts14**, **Adamts15**, **Adamts18**, **Adamts19**	Suppl. Figure 2f
Chemokine ligands (CCL)^^*^)^	**Ccl1**, Ccl3^*^, **Ccl4**, Ccl5^*^, Ccl6^*^, **Ccl9**, Ccl11, Ccl12^*^, **Ccl17**, **Ccl21**, Ccl25, Ccl27, Ccl28	Ccl2^*^, **Ccl5**, Ccl7^*^, Ccl9, Ccl11, Ccl12, Ccl17, Ccl20^^*^)^, Ccl21, Ccl22, **Ccl24**, Ccl25, Ccl27, Ccl28	Suppl. Figure 3a
C-C-chemokine receptor (CCR) / C-C-chemokine ligand (CCRL)	**Ccr1**, Ccr1l1^b)^, Ccr2^b)^, Ccr3^b)^, Ccr4^b)^, Ccr5^*^^b)^, **Ccr6**, Ccr9^b)^	Ccr1l1^b)^, Ccr2^b)^, **Ccrl2**, Ccr3^b)^, Ccr4^b)^, Ccr5^b)^, **Ccr7**, Ccr9^b)^, Ccr10	Suppl. Figure 3b
CXC-Motive-chemokine receptor (CXCR)	Cxcr1^b)^, Cxcr2^b)^, Cxcr3^b)^, **Cxcr4**, Cxcr5, Cxcr6^b)^, Cxcr7^b)^	Cxcr1^b)^, Cxcr2^b)^, Cxcr3^b)^, Cxcr6^b)^, Cxcr7^b)^	Suppl. Figure 3c
Transforming Growth Factor (TGF)	TGFβi^*^, **TGFβR1**^b)^, **TGFβR2**^b)^, **TGFβR3**^b)^	**TGFα, TGFβ1**, TGFβ2^*^, **TGFβ3**, **TGFβR1**^b)^, TGFβR2^b)^, TGFβR3^b)^	Suppl. Figure 3d

^a)^ upregulation/downregulation defined as 1.1fold modulation versus control cells growing *in vitro*, bold gene abbreviations/names indicate >twofold modulation

^b)^ genes, which show a biphasic modulation, are listed both, under upregulation and downregulation

^*)^ an asterisk indicates >tenfold modulation versus control cells growing *in vitro*

The proteoglycans perlecan and neurocan didn't show significant modulation during liver colonization,­ while versican and aggrican were significantly decreased in expression during all stages (supplementary Figure 2c).

Gene products, which alter the cellular environment, include the metalloproteinases. From 20 MMP members, 6 (30%) and 3 (15%) were significantly up- and downregulated (supplementary Figure 2d). MMP2, MMP12, and MMP23 were ≥ 9fold up-regulated during terminal colonization (21 days). MMP23 was initially expressed at a low level but increased dramatically in the terminal stage. MMP2 expression increased­ gradually from first till last stage. MMP19 and MMP24 were significantly down-regulated­ in the terminal stage of colonization. Tissue inhibitors of metalloproteinases (Timps) restrict MMP activities. Here, only one of four Timps (Timp1) was significantly decreased in expression.

From the zinc proteases termed “A disintegrin and metalloproteinase domain” (Adam), 16 of 25 family members (64%) were modulated in expression. The minority showed increased expression, such as Adam18 and Adam20, but the majority showed reduced expression, such as Adam6, Adam15, Adam19, and Adam23 (supplementary Figure 2e). In the subfamily Adamts (ts = thrombospondin motifs), Adamts4 and Adamts9 were decreased in expression during liver colonization whereas Adamts1 and Adamts2 were increased in expression,­ especially during early and terminal stages (supplementary Figure 2f).

Chemokines are secreted and act in the ECM, they are involved in the growth of many cancers and facilitate immune evasion, tumour progression and metastasis. For functional reasons chemokine­ receptors, which are part of the ECM with their N-terminal part only, were included in this analysis. The expression of many chemokine families showed a most impressive alteration during liver metastasis. Eleven of 28 chemokine genes (39%) were modulated in ASML cells during liver colonization, with seven being significantly up-regulated and four significantly down-regulated (supplementary Figure 3a, 3b, 3c).

Transforming growth factor (TGF) genes are multifunctional regulatory polypeptides that are prototypical members of a large family of cytokines controlling many aspects of cellular functions­. TGFβi was ≥ 20fold up-regulated during terminal stage of colonization. In addition, TGFβR2 and TGFβR3 were significantly­ increased. By contrast, TGFβ1, TGFβ2 and TGFβ3 were significantly decreased (supplementary Figure 3d).

### Confirmation of microarray results by qRT-PCR, q-PCR, and Western blot

**Figure 5:**
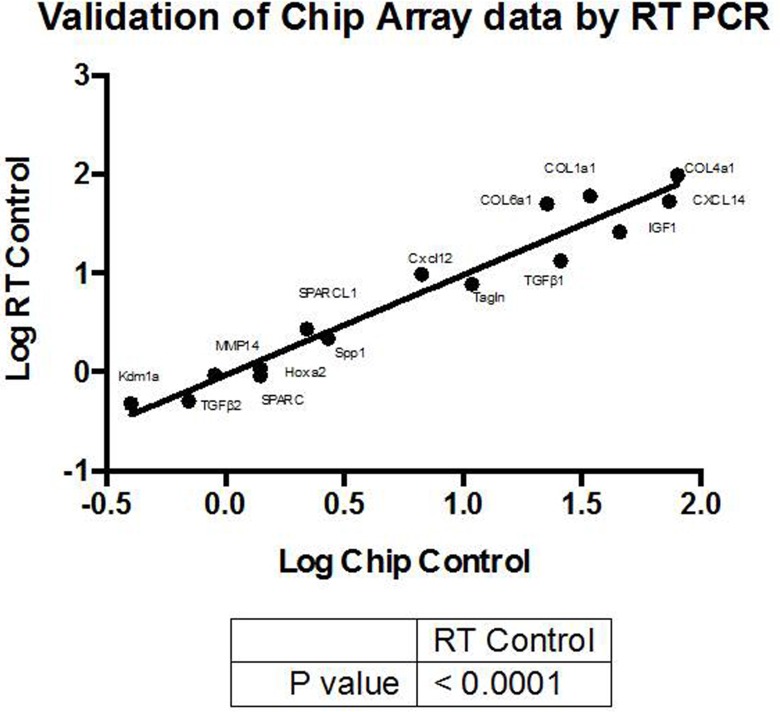
The expression of 15 genes by either microarray (Χ-axis) or RT-PCR (Υ-axis) was correlated by trend line The resulting was *p* ≤ 0.001. For full gene designations see Supplementary Table 1.

Quantitative RT-PCR as well as Western blot was used to confirm the *in vivo* expression profile of selected genes. Overall, the correlation between microarray and RT-PCR was very good as shown by the corresponding p value obtained from 15 genes (*p* ≤ 00.01 see Figure 5). The range of fold changes spanned over more than two logs and thus corresponded closely to the difference in gene expression, which can be detected by microarray (103). In addition, the relationship between q-PCR and western blot was also satisfying as shown for 6 genes (see supplementary Figure 4).

### Knockdown of Mmp2 and Ccl20

**Figure 6:**
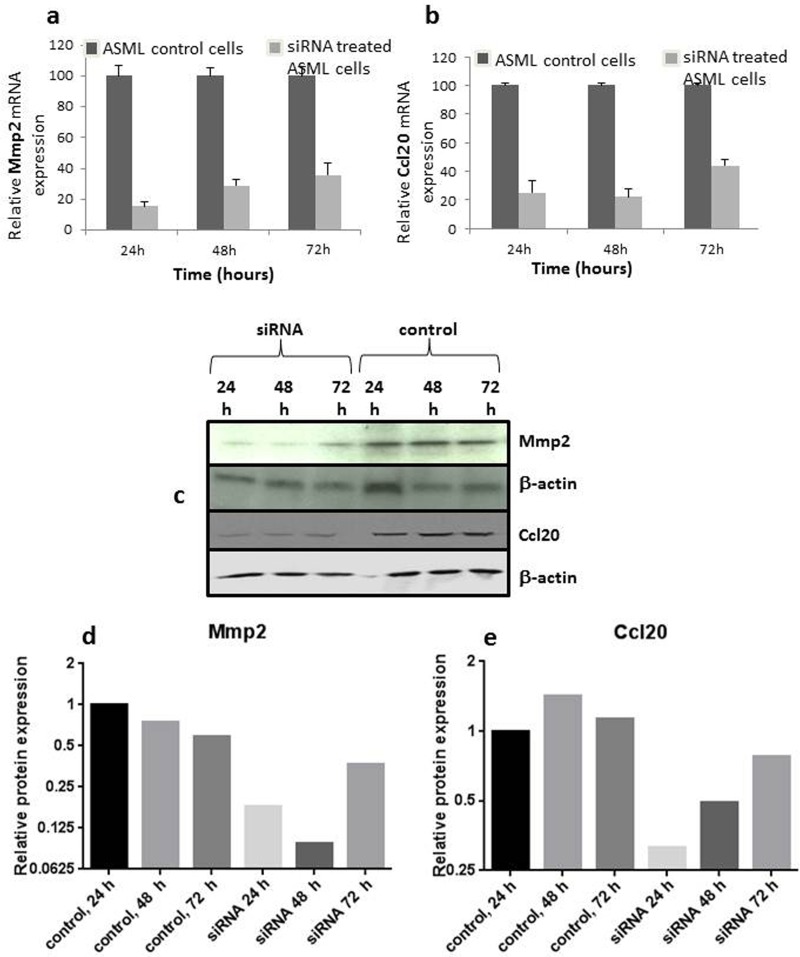
Knockdown of Mmp2 and Ccl20 by specific siRNA oligomers The upper row shows the relative mRNA expression following transfection with siRNA after 24 to 72h for Mmp2 **a.** and Ccl20 **b.** The middle **c.** and bottom **d.**, **e.** rows show the corresponding protein levels as determined by Western blot. For graphical presentation, the bands were analyzed by Image J pro­gram and the band intensity was normalized with regard to the respective actin levels.

**Figure 7:**
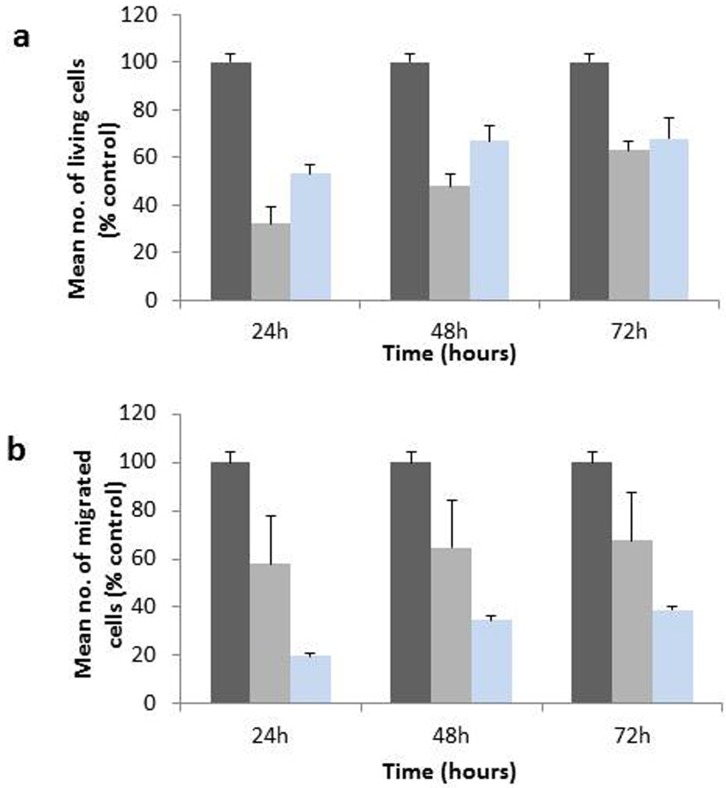
Proliferation and migration of ASML cells following knockdown by siRNA oligo­mers against Mmp2 and Ccl20 Black columns represent untreated control cells, grey columns indicate the effect of knockdown by siRNA against Ccl20, blue columns that by siRNA against Mmp2. **a.** ASML cell proliferation was determined by MTT assay over 72 h. **b.** Migration of ASML cells was followed for 72 h.

Knockdown by siRNA of Mmp2 and Ccl20, which were 32fold up- and 1000fold downregulated *in vivo*, respectively, was used to address the question, whether the modulation of these genes contributed causally to the process of liver colonization. It was tempting to choose Mmp2 and Ccl20 as targets, because the gene groups, which they represent, have been of therapeutic interest before. As shown in supplementary Figure 5, the tumour tissue of a lesion, which was excised at 9 days after ASML PDAC cell implantation showed increased expression of Mmp2 and decreased expression of Ccl20 when compared to cells grown under cell culture conditions. Transfection of specific siRNA oligomers expectedly decreased the mRNA and protein expression of Mmp2 and Ccl20 in ASML cells for up to 72 h (see Figure 6a-6e). ASML cells transfected with these siRNA oligomers showed reduced survival as verified by MTT test (Figure 7a) as well as reduced migration­ as shown by Boyden chamber assay (Figure 7b). Interestingly, knockdown of Mmp2 had a lower effect on cell proliferation and a higher inhibition of migration than observed for the corresponding­ knockdown of Ccl20.

## DISCUSSION

PDAC is characterized by early metastasis and late detection of the primary tumour, as 80% of patients present at an advanced stage, i.e. with metastases at their first visit to a doctor. Most of these metastases arise in the liver. Therefore, monitoring of genes which are differentially expressed­ during liver metastasis may aid in the identification of novel strategies to detect and treat this highly lethal disease [1].

Our analysis of ASML PDAC cells colonizing the rat liver showed that 49 % of all genes were at least twofold modulated in expression within the first three days after intraportal injection. At this early stage, there was a huge preponderance of up-regulated over down regulated genes (≈20:1). The activation of almost half of the whole genome is probably related to the new environment since ASML cells started interacting with the respective ECM of the rat anatomy instead of a plastic surface. The unexpected imbalance between genes with increased and decreased expression resembles an arousal reaction and probably indicates a general activation of a large number of genetic materials with no specific task in the colonization process. Thus we assumed that the genes responsible for progression would be better recognized after the ‘noise’ of modulated gene expression had decreased. In line with this, the ratio of up- versus down-regulated genes decreased over 10fold at all later stages. Concomitantly, the total number of at least 2fold modulated genes was halved. Classification of these genes into functions which are related to neoplastic growth showed that the initial rise in gene expression was not by chance but fol­lowed a teleological strategy. This is exemplified by our findings that ‘migration and movement’ as well as ‘proliferation’ of ASML cells were significantly increased from early on but not ‘angi­ogenesis/vascularization’, which was up-regulated only during the terminal stage of liver coloni­ zation. A comparison of the four stages showed further that the intermediate stage was related to the lowest number of modulated genes, and this was associated with decreased activation of ‘migration­ and movement’ as well as ‘proliferation’ of ASML cells. Based on this information, the intermediate stage can be regarded as potential bottle neck of liver colonization and respective targets of anti-metastatic treatment could possibly be exploited most beneficial during this stage. The formal analysis of gene modulations, which were based on the time when ASML cells grew in rat liver as well as the period of in vitro growth thereafter, showed that the environment dis­tinctly influenced mRNA expression. We then concentrated on the expression of genes, which are instrumental in shaping the environment of cells and focused on those contributing to the ECM.

Within the group of structural ECM proteins, collagens showed the most intensive variation in expression, ranging from 100fold increased to 20fold reduced levels as compared to their *in vitro* profile. At first, this indicates that PDAC cells shape their environment by increasing the availability­ of certain collagens, while decreasing the level of others. Concurrently, certain Mmps were up-regulated, such as Mmp2, Mmp12, Mmp23 and Mmp7, whereas others were down-regulated like Mmp11, Mmp19 and Mmp24. Downregulation of Mmp2 by siRNA *in vitro* was associated with reduced proliferation and migration in respective cell culture assays, indicating that reduced expression of Mmp2 *in vivo* might hamper liver colonization. The increased availability of Mmp2 and Mmp12 as well as their substrates Col1a1, Col4a1, Col6a1 and LamC2 could indicate that the tumour cells generated foremost collagens, which can be cleaved by these Mmps. In fact, breakdown of collagens by Mmps will generate proline, which constitutes more than 25% of residues in collagen [16]. Recent studies document an important role of proline catabolism to meet the needs of proliferation and/or survival during tumour progression [17, 18]. Proline has its own metabolic enzymes; it is catabolized to pyrroline-5-carboxylate by proline oxidase (POX). Under conditions of hypoxia, POX mediated proline metabolism can function as autophagy signalling. Furthermore, POX is a downstream target of p53, but if p53 has lost control, POX will generate proline-dependent ROS thus favouring tumour cell persistence and tumour progression[19]. The increased production of certain collagens and Mmps appears to be instrumental in fuelling the shape of an ECM, which favours the survival and progression of PDAC cells. Regulation of these changes in expression is achieved by miRNAs, which control a varying series of dependent­ mRNAs [20, 21]. In case of miR29-3p, 7 collagen genes are known to be regulated. Loss of miR29 was reported to be correlated with a significant increase in ECM deposition [22]. We found a moderate to pronounced decrease in miR29-3p levels, which was related to increased mRNA expression of Col1a1, Col1a2, Col3a1, Col4a1, Col4a2, Col5a2, and Col5a3. Several studies have demonstrated that high expression of miR-29 induces apoptosis in hepatocellular carcinoma cells, while down-regulation of miR-29 increases liver fibrosis, HCC tumorigenicity and metastasis [22–24]. Further, miR-29 hinders cancer progression by endorsing tumour cell apoptosis,­ by suppressing DNA methylation of tumour-suppressor genes, and by decreasing proliferation­ of tumours [25, 26]. However, the level of miRNA29b-3p found in this study does not fully reflect the changes in mRNA levels of dependent genes. In the case of Col1a2 it is obvious that the terminal increase in expression of this gene's mRNA can be better explained by co-regulation­ resulting from both, miR29b-3p and miR let-7a-5p.

Chemokines are powerful means of cell regulation, which are increased during inflammation. As cancer can be considered a chronic inflammation, it is not too surprising that ASML cells used these signalling molecules for their purposes. For having an overview of the system, we included the chemokine receptors, although they are part of ECM with their N-terminal moiety, only. As for collagens, there was a tremendous modulation of mRNA expression, ranging from a 100fold increased to 1000fold decreased expression levels (Ccl3, Ccl20, respectively). Chemokine Ccl20 functions as chemoattractant and pairs with only one receptor, which is Ccr6. Knockdown of Ccl20 by siRNA caused reduced proliferation and migration in ASML cells growing *in vitro*. Currently it is unclear, why ASML cells downregulated Ccl20 by more than three orders of magnitude­. Reasons could include that the tumour cells try to reduce stimuli, which are active in epithelial­-mesenchymal transition [27], as they are to colonize an organ, as well as to avoid stimulation­ of immune cells. In this context, many roles in immune cells have been reported [28]. Both molecules were significantly up-regulated in PDAC as well as several other cancer types and were reported to promote cancer cell proliferation and migration. Moreover, CCL20/CCR6 apparently­ plays a role in organ selective liver metastasis of colorectal cancer [29–31]. In this study the ligand Ccl20 was drastically reduced in expression, whereas its receptor was just modestly up-regulated. It may therefore be hypothesized that the shutdown of Ccl20 is related to the poor immunogenicity of ASML cells. In variance to Ccl20/Ccr6, several ligands, including Ccl3, bind to Ccr1 and Ccr5. Both chemokine receptors play a crucial role in the migration and metastasis of human cancers [32–34]. In this experiment Ccl3 as well as Ccr1 and Ccr5 were distinctly increased­ in expression. These increases indicate that the chemokine and its receptors can be considered­ a target of anti-metastatic therapy. Recent studies exploring this concept at translational and clinical levels have shown startling results in terms of tumour cell inhibition in experimental systems as well as a recent Phase I clinical study involving maraviroc, an inhibitor of Ccr5, as therapeutic agent [35–38].

Members of the TGFβ family are multifunctional cytokines, which have been implicated in nearly all key steps of tumorigenesis [39]. TGFβ acts as tumour suppressor in early pancreatic carcinogenesis,­ due to its growth-inhibitory effect in epithelial cells, but in advanced disease appears to promote tumour progression and metastasis, presumably because it can induce epithelial to mesenchymal transition [40–42]. A study in colorectal cancer showed that distinct expression of transforming growth factor-β induced (TGFβI) in cytoplasm and stroma was correlated with lymph node and distant metastasis [43]. TGFβI serves as a linker protein, which mediates integrin binding to the ECM proteins such as collagen, laminin and fibronectin and has a role in the activation­ of morphogenesis, cell proliferation, adhesion, migration, differentiation and inflammation­. In addition, high TGFβI expression is a powerful biomarker of poor prognosis in cancer [44–46]. In our data the expression of TGFβI, was most clearly increased, followed by the mRNAs of TGFβR2 and TGFβR3. In contrast, TGFα as well as TGFβ1, 2, 3 were all decreased in mRNA expression. We hypothesize that ASML cells are resistant to the growth inhibitory function of TGFβ, and the reduced expression of these cytokines prompted an upregulation of the respective receptors in order to make up for the loss in signal strength. We furthermore speculate that the distinct increase in TGFβI mRNA is to be correlated with increased levels of members of the collagen and laminin families as well as with increased Mmp-2 secretion [45, 46].

The results and conclusions outlined above are valid, of course, primarily in their experimental context and based on the methods used. Therefore, some thoughts about possible limitations seem to be appropriate. Limitations of the rat model include e.g. the rapid rate of liver colonization and the young age of rats during this process, which differ from the respective observations in PDAC patients [2]. In addition, the use of an mRNA microarray as opposed to RNA sequencing was based on our choice for an affordable and robust test, especially since we did not know which genes we wanted to analyse, and because we did a whole transcriptome analysis of differentially expressed genes on the basis of a good reference sequence for rats. These advantages as well as the relatively low amount of raw data, which can be accessed by well-established and easy-to-use software packages, outweighed the low dynamic range of the micro-array technique. In addition, we decided to examine DNA variations (SNPs, insertions, deletions) and genes with low abundance as well as to possibly discover new genes or alternative splice variations at a later point in time [47].

In summary, the changes in mRNA expression detailed in our experiment are promising for identifying­ potential targets of anti-metastatic treatment as well as biomarkers, which can be used for early diagnosis. Further experiments will concentrate on exploiting these findings.

## MATERIALS AND METHODS

### Cell lines and culture conditions

ASML cells were maintained in RPMI-1640 medium (Invitrogen), supplemented­ with 10% foetal bovine serum (FBS), L-glutamine (2mM), penicillin (100 IU/ml), and streptomycin (100μg/ml). The cells were kept at 37 °C in a humidi­fied incubator with an atmosphere of 5% CO2 in air. For isolation and propa­gation,­ cells were washed with phosphate buffered saline (PBS), trypsinised (0.25% trypsin/EDTA) and pelleted at 1500 rpm for 5min. By seeding appropriate cell numbers into new flasks, the cells were maintained in logarithmic growth.

### Animal experiments

6-8 weeks-old male BDX rats were obtained from the German cancer research center animal facility­ at a body weight of 150-190 g. The rats were kept under specified pathogen free (SPF) conditions­ (23°C ± 1°C temperature, 50% ± 10% humidity and a 12h dark-light rhythm) and housed in macrolon III cages with external ventilation by filtered air (Ventirack, UN Roestvaststaal, Zevenaar, Netherlands). The rats were given an adaptation period of 1 week prior to any experimental­ procedure.

### Tumour cell transplantation

Logarithmically growing ASML_GFP-Luc_ cells (2 × 106 cells) were implanted into the rats' portal vein as described before [10, 11]. Animals harbouring these cells in their liver were kept for various­ periods until tumour cell re-isolation.

### ASML tumour cell re-isolation

The experiment was designed to investigate the temporal changes in gene expression, which take place in ASML tumour cells colonizing the rat liver, as compared to control cells. The gene modulations­ were identified by microarray analysis from tumour cells isolated from whole rat livers, to avoid sampling biases. For this purpose, five different time points (1, 3, 6, 15 and 21 days) after tumour cell implantation were chosen for re-isolation of ASML cells. Altogether 10 rats (2 per time point) were used for the experiment. In addition, 2 rats were used respectively, on days 1, 3, 6 and 15 after tumour cell implantation for histologic evaluation of rat liver infiltration by tumour cells. To obtain tumour cells of high purity, the ASML cells were isolated by fluorescence activated cell sorting (FACS) using green fluorescent protein (GFP) as marker. The purity of these ASML_GFP-Luc_ cell preparations was greater than 99%. The number of cells isolated for cDNA preparation ranged from 105 (minimum) to 10_8_ cells per liver. Afterwards, the pure ASML cells were pelleted at 3,000 rpm for 5 min and kept at −80 °C. For further experiments, the ASML_GFP-Luc_ cells resulting from two rats per time point were pooled. The animal experiment was approved­ by the responsible authority (Regierungspräsidium Karlsruhe) in full compliance with the German animal welfare protection law.

### Histopathologic evaluation

Rat livers were fixed in formaldehyde (4%, in PBS), embedded in paraffin, and cut in 4-µm thick serial sections. Tissue sections were mounted onto adhesive microscope slides, stained with haematoxylin-eosin (H&E) and examined by an experienced pathologist (IB) for tumour cell infiltration.

### RNA Isolation and cDNA syntheses

RNA-isolation from ASML cells was performed as described before. After producing cDNA from the isolated RNA, amplicons of cDNA were generated with the respective primers for selected­ genes as shown in Supplementary Table 1. Expression of γ-tubulin2 was used for normalizing the target gen levels. The PCR-reactions and Gel Doc analyses were performed as described before [11, 48].

### Quantitative real-time RT-PCR

Expression profiles of ASML cells were studied by qRT-PCR methodology. For this purpose, cDNA (1μg) from control and liver tumour samples was subjected to PCR amplification by using 2× LC480 master mix along with an appropriate probe from the Universal probe library (Roche, Mannheim, Germany). Samples were processed in triplicate, and the expression level of γ-tubulin was used for normalization of the data. The fold-changes in the expression levels were calculated by the 2-ΔΔCT method.

### Western blot

Isolated ASML cells were lysed, separated by electrophoresis and blotted as described before [48]. PVDF membranes were probed for different proteins (CcL2, Ccl20, Mmp2, Mmp7, Mmp14 and β-actin) using specific antibodies (Santa Cruz, Biotechnology, Germany) as per manufacturer's­ instructions. Immunoblots were developed using conjugated anti-mouse or anti-rabbit IgG (Santa Cruz, Biotechnology, Germany) and ECL-System (Amersham Pharmacia Biotech, Munich,­ Germany). Levels of β-actin were used to normalize the protein expressions. Relative concentrations­ were assessed from radiographic images using the ImageJ Program.

### Microarray

Messenger RNA expression analysis was done by Illumina Rat Sentrix-8 Bead Chip arrays (Illumina,­ San Diego). 30,508 gene probes and 1,776 miRNA probes are contained in this Illumina product. The microarray was performed as described before [11]. Data analysis was done for all beads individually.

### siRNA transfection of ASML pancreatic cancer cells

ASML cells were plated overnight at a density of 200 000 cells per well in six-well plates. A total of 100 µl transfection solution containing 6 ng (final concentration 100-200 nM) of siRNA oligomers­ directed against Ccl20 (GCUGCCUCACGUACACAAA) or Mmp2 (GGAAACCAA­GAUGUGGCAA), or negative control siRNA (Invitrogen, Karlsruhe, Germany) and 15 µl trans­ fection reagent (Invitrogen) were added to 1.9 ml medium per well. After 24 h, cells were trypsinized­ and used for subsequent protein or mRNA extraction, for immunoblot analysis or for MTT and migration assays.

### MTT assay

To assess the effect of siRNA transfection on the proliferation of ASML cells, the 3-(4, 5-dimethylthiazol­-2-yl)-2,5-diphenyltetrazolium bromide (MTT) assay was used as described earlier [49].

### Cell migration

This assay was performed to investigate the effect of Ccl20 or Mmp2 down-regulation on the migration of ASML cells. The bottom layer in 24-well plates consisted of 50 µl FCS, which was gently over-layered with 200 µl semi-liquid RPMI medium (containing 0.4% methylcellulose and 20% FCS) resulting in the chemotaxis mixture. An incubation time of 24 h was needed to build the chemotaxis gradient. Then, ASML cells were seeded on 8 µm pore size polycarbonate membranes­ (Millicell; Millipore, Schwalbach, Germany), which were transferred onto the prepared wells. The next day, ASML cells were transfected with nonsense or specific siRNA against Ccl20 or Mmp2 as described before. Migrating cells were counted under a microscope for 3 subsequent days. The membrane with transfected, non-migrated cells was transferred daily onto a fresh well with chemotaxis gradient.

### Statistics

Quantile-normalized mRNA and miRNA data were log2 transformed. Differentially expressed transcripts between groups were identified with the empirical Bayes approach based on moderated­ t-statistics as implemented in the Bioconductor package limma, *P*-values ≤ 0.05 were considered­ significant. Pairing of samples was accounted for as appropriate [50].

### Ingenuity

The data were also analysed by Ingenuity Pathways Analysis (IPA; Ingenuity® Systems, www.ingenuity.com). A data set having gene identifiers and corresponding expression values was uploaded into in the IPA application. Each gene identifier was mapped to its corresponding gene object in the Ingenuity Pathways Knowledge Base. A fold change cut off of 2 was set to identify genes the expression of which was significantly differentially regulated. These genes, called focus genes, were overlaid onto a global molecular network developed from information contained in the Ingenuity Pathways Knowledge Base. Networks of these focus genes were then algorithmically generated based on their connectivity.

## SUPPLEMENTARY FIGURE AND TABLE



## Abbreviations

PDAC: Pancreatic ductal adenocarcinoma
ECM: Extracellular matrix
miRna: Micro RNA
FACSF: luorescence-activated cell sorting
SPF: Specified pathogen free
IPA: Ingenuity Pathways Analysis
Col: Collagen
Mbp: Myelin basic protein
Vtcn1: V-set domain containing t-cell activation inhibitor 1
Mao-A: Monoamine oxidase A
Nr2f4: Nuclear receptor subfamily 2 group f member 1
Epha8: Eph Receptor A8
Cacna1b: Calcium voltage-gated channel subunit alpha1 B
Sdcbp2: Syndecan binding protein 2
Olr85: Olfactory receptor 85
Mmp: Metalloprotease
Timp: Tissue inhibitor of metalloproteinase
Adams: A disintegrin and metalloproteinase domain
Adam-ts: Adam-thrombospondin motif
TGF: Transforming growth factor
Vtn: Vitronectin
Fn: Fibronectin
Nid-1: Nidogen1/entactin
Hapln: Hyaluronan and proteoglycan link proteins
Habp: Hyaluronan binding protein
