# Supplementary material for: Pancreatic carcinoma cells colonizing the liver modulate the expression of their extracellular matrix genes
Source: Genes Cancer. 2018 May;9(5-6):215–31. doi: 10.18632/genesandcancer.179 (PMC6305105; doi:10.18632/genesandcancer.179)
Supplement: Supplementary file 1 [file ganc-09-215-s001.pdf]

Pancreatic carcinoma cells colonizing the liver modulate the expression of their extracellular matrix genes - Al-Taee et al

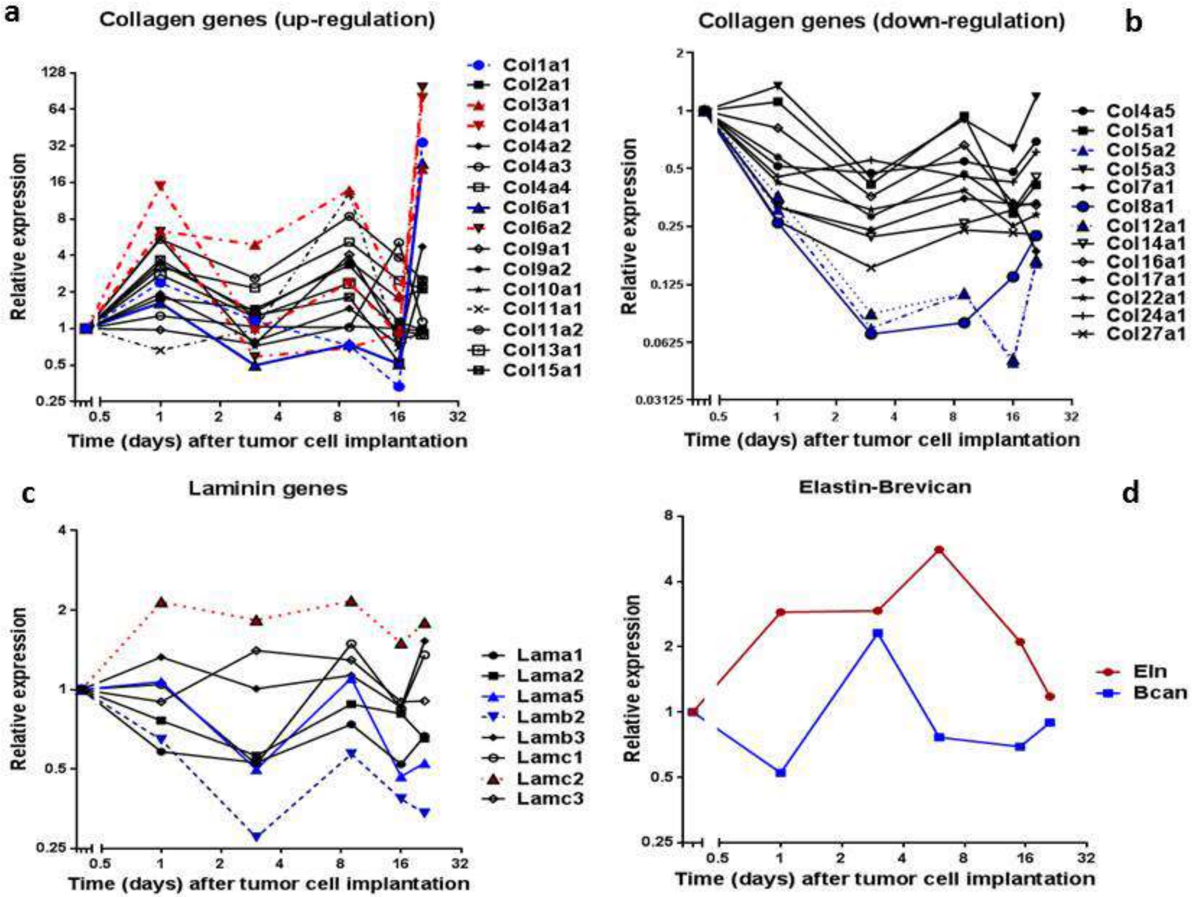

Suppl. Figure 1: Expression of structural ECM genes in ASML cells during and after liver colonization. The values in the diagram represent the gene expression in re-isolated (1, 3, 6, 15, 21 days) ASML cells in comparison to the expression of ASML cells growing in vitro, set to unity. (a, b) Expression of collagens; (c) Expression of laminins; (d) Expression of elastin and brevican.
Red colors indicate significantly increased expression; blue colors indicate significantly decreased expression, black color indicates genes with no significant modulation.

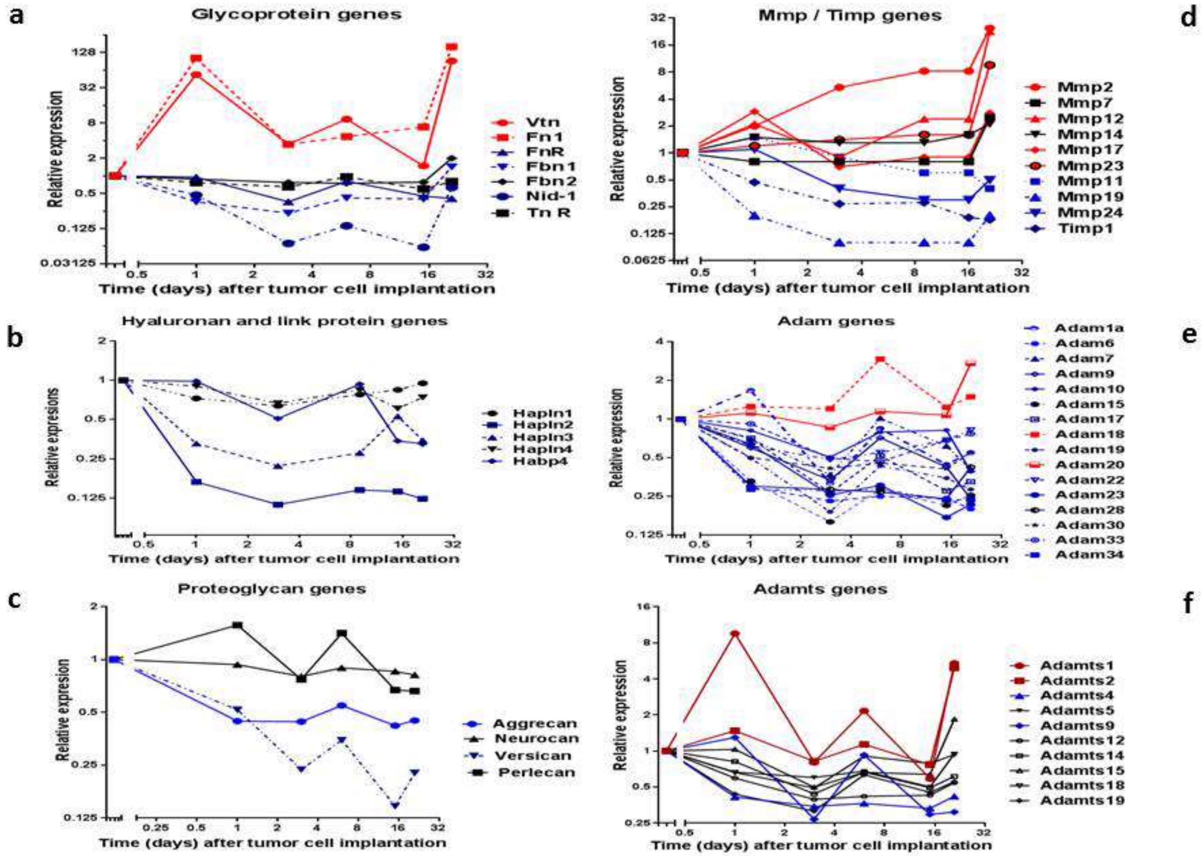

Suppl. Figure 2: Overview of expression modulation during rat liver colonization for glycoprotein genes (a), hyaluronan and link protein genes (b), proteoglycan genes (c), metalloprotease / tissue inhibitor of metalloproteinase genes (d), a disintegrin and metalloproteinase domain genes (e), and adam-thrombospondin motif genes (f).

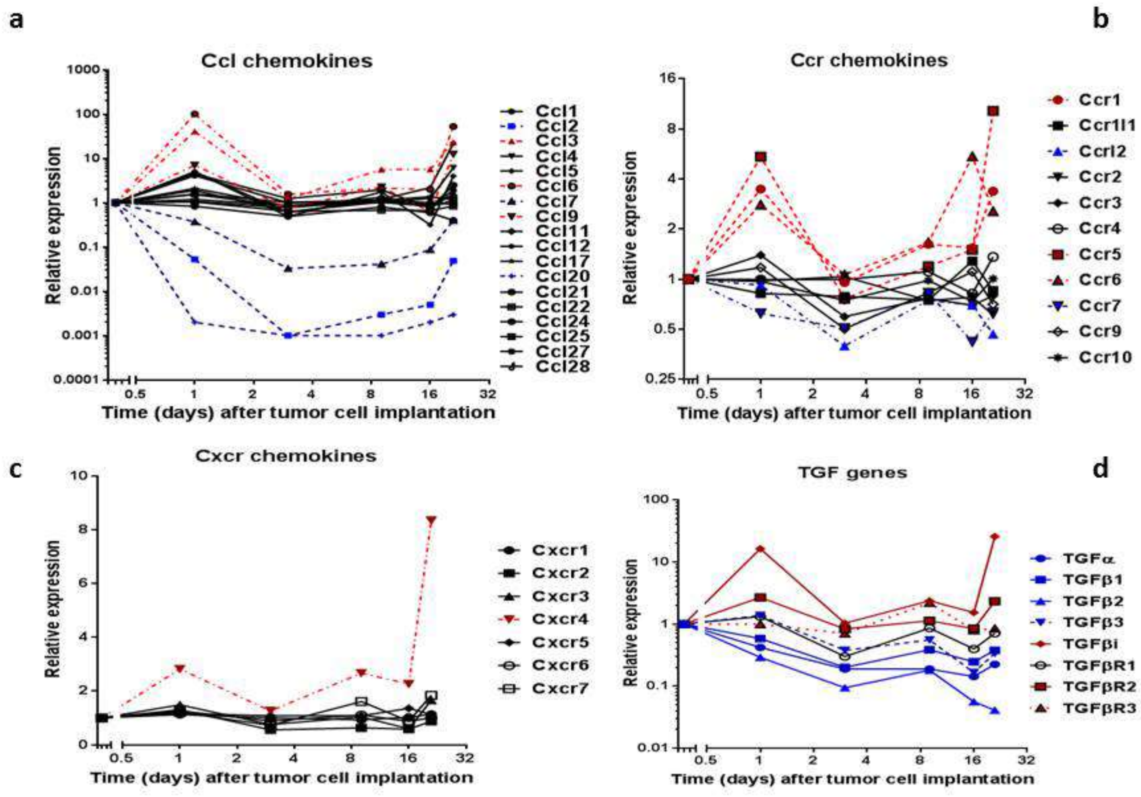

Suppl. Figure 3: Expression of chemokines, chemokine receptors and TGF family genes in ASML cells during and after liver colonization. (a) Ccl chemokines, (b) CCr chemokines, (c) Cxcr chemokines,(d)TGF genes.

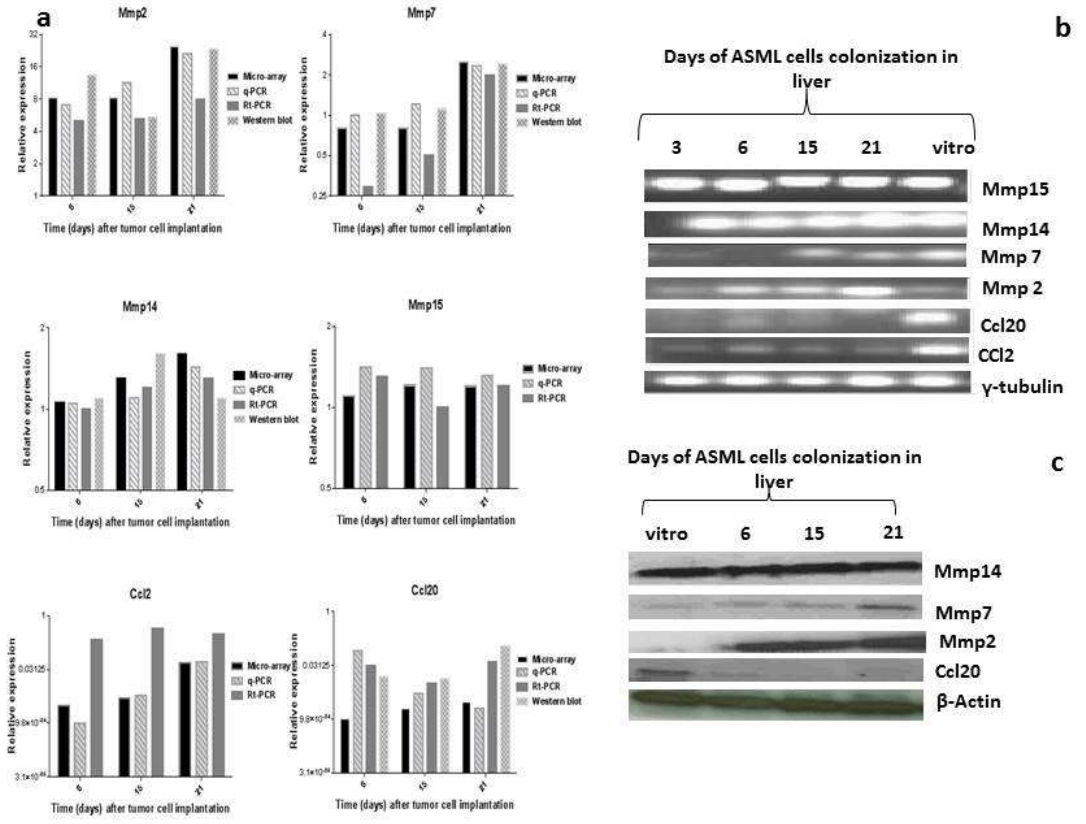

Suppl. Figure 4: (a) Expression profiles of Mmp2, Mmp7, Mmp14, Mmp15, CCL2, and CCL20, as shown by microarray analysis, qRT-PCR, RT-PCR and western blot. (b) The values represent the gene expression by qRT-PCR in re-isolated metastasizing cells in comparison to the expression of cells growing in vitro for Mmp2, Mmp7, Mmp14, Mmp15, CCL2, and CCL20. (c) Protein expression of selected genes by Western blot.

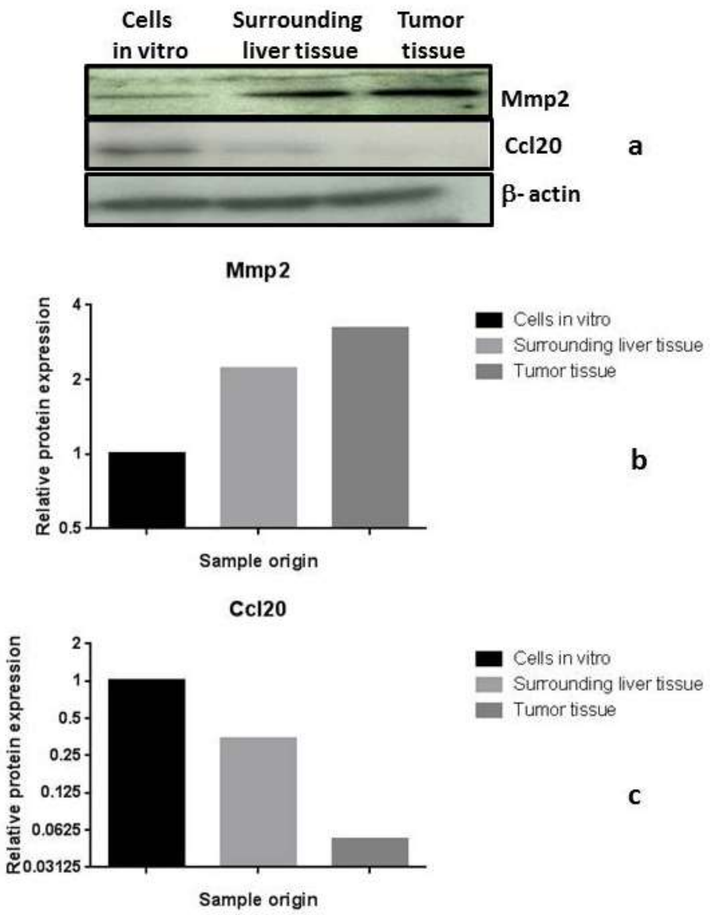

Suppl. Figure 5: Western blot of ASML PDAC cell samples originating from in vitro (cell culture) or in vivo (liver tissue surrounding a tumor nodule and a tumor nodule excised at 9 days after tumor cell implantation). (a,) The protein bands of Mmp2 and Ccl20 show a clear increase in Mmp2 level when comparing in vitro versus in vivo condition (b) and similarly a clear decrease for Ccl20 (c). Interestingly, healthy liver tissue surrounding the tumor nodule shows an intermediate expression level of the two proteins.

Suppl. Table 1: Overview on selected genes and respective rat primer sequences

Gene designation	Gene abbreviation	Species	Left primer	Right primer	ID probe
Matrix metallopeptidase 2	Mmp2	rat	caccaccgaggattatgacc	cacccacagtggacatagca	04693477001
Matrix metallopeptidase 7	Mmp7	rat	gtactggacggatggtgagg	aactgggaacagaagagtgacc	04693493001
Matrix metallopeptidase 14	Mmp14	rat	aacttcgtgttgcctgatga	tttgtgggtgaccctgactt	04688015001
Matrix metallopeptidase 15	Mmp15	rat	gaagacgccgaagtatacgc	gctggggtaggtagccataga	04688945001
C-C motif chemokine ligand 2	Ccl2	rat	agcatccacgtgctgtctc	gatcatcttgccagtgaatgag	04688619001
Chemokine (C-C motif) ligand 20	Ccl20	rat	tctgcccttcctggcttt	gcagtcaaagttgcttgcttc	04688945001
Tubulin, gamma 2	-tubulin2	rat	caactccctcctcaccctaa	gttcagggccgtgttgtc	04693493001
Collagen, type VI, alpha 1	Col6a1	rat	aagagaggggcctgttgg	ggtatcctttaggtccgatgg	04689038001
Collagen, type IV, alpha 1	Col4a1	rat	ccatggtcaggacttgggta	aagggcatggtgctgaact	04693469001
Collagen, type I, alpha 1	Col1a1	rat	aggtccccctggaaagaa	aggtccccctggaaagaa	04689020001
Chemokine (C-X-C motif) ligand 14	Cxcl14	rat	cctatcccaaccaggcaag	cctcagaggttgcgagagaa	04686900001
Lysine (K)-specific demethylase 1A	Kdm1a	rat	ttctggagggtatggagacg	gcttcctgagaggtcattcg	04688589001
Insulin-like growth factor 1 transcript variant 2	Igf1	rat	catgcccaagactcagaagg	cgtggcattttctgttcctc	04688627001
Transforming growth factor, beta 1	Tgfb1	rat	cctggaaagggctcaacac	tgccgtacacagcagttctt	04684974001
Transforming growth factor, beta 2	Tgfb2	rat	gacatgccgtcccacttc	cactgagccagaggatgttg	04693469001
C-X-C motif chemokine ligand 12, transcript variant 1	Cxcl12	rat	agtgtggccctgatgtgg	tcaccaacttgctcagaatca	04685024001
Secreted protein acidic and cysteine rich	Sparc	rat	ccctgccgattctttgag	tgttgcttttcagccttgc	04686942001
Secreted phosphoprotein 1	Spp1	rat	ggaagctgcagaagagatgg	ggttggcacccacaggta	04686993001
Homeo box A2	Hoxa2	rat	agaaggcggccaagaaaa	cggctatttccagggattct	04688937001
Matrix metallopeptidase 14	Mmp14	rat	aacttcgtgttgcctgatga	tttgtgggtgaccctgactt	04688015001
SPARC-like 1	Hevin	rat	gaggggattgaacttcagctt	ccttcatgctggctagacct	04688635001
